# Field evaluation of transgenic hybrid poplars with desirable wood properties and enhanced growth for biofuel production by bicistronic expression of *PdGA20ox1* and *PtrMYB3* in wood-forming tissue

**DOI:** 10.1186/s13068-021-02029-2

**Published:** 2021-09-07

**Authors:** Jin-Seong Cho, Min-Ha Kim, Eun-Kyung Bae, Young-Im Choi, Hyung-Woo Jeon, Kyung-Hwan Han, Jae-Heung Ko

**Affiliations:** 1 Department of Plant & Environmental New Resources, Kyung Hee University, 446-701 Yongin, Republic of Korea; 2 Division of Forest Biotechnology, National Institute of Forest Science, 441-847 Suwon, Republic of Korea; 3 Department of Horticulture and Department of Forestry, Michigan State University, East Lansing, MI 48824-1222 USA; 4 Present Address: Abio Materials Co., Ltd, Cheonan, 31005 Republic of Korea

**Keywords:** Bicistronic gene expression, Biofuel, Developing xylem promoter, Hybrid poplar, LMO field experiment, PdGA20ox1, PtrMYB3, Saccharification

## Abstract

**Background:**

To create an ideotype woody bioenergy crop with desirable growth and biomass properties, we utilized the viral 2A-meidated bicistronic expression strategy to express both *PtrMYB3* (*MYB46* ortholog of *Populus trichocarpa*, a master regulator of secondary wall biosynthesis) and *PdGA20ox1* (a GA20-oxidase from *Pinus densiflora* that produces gibberellins) in wood-forming tissue (i.e., developing xylem).

**Results:**

Transgenic *Arabidopsis* plants expressing the gene construct DX15::PdGA20ox1-2A-PtrMYB3 showed a significant increase in both stem fresh weight (threefold) and secondary wall thickening (1.27-fold) relative to wild-type (WT) plants. Transgenic poplars harboring the same gene construct grown in a greenhouse for 60 days had a stem fresh weight up to 2.6-fold greater than that of WT plants. In a living modified organism (LMO) field test conducted for 3 months of active growing season, the stem height and diameter growth of the transgenic poplars were 1.7- and 1.6-fold higher than those of WT plants, respectively, with minimal adverse growth defects. Although no significant changes in secondary wall thickening of the stem tissue of the transgenic poplars were observed, cellulose content was increased up to 14.4 wt% compared to WT, resulting in improved saccharification efficiency of the transgenic poplars. Moreover, enhanced woody biomass production by the transgenic poplars was further validated by re-planting in the same LMO field for additional two growing seasons.

**Conclusions:**

Taken together, these results show considerably enhanced wood formation of our transgenic poplars, with improved wood quality for biofuel production.

**Supplementary Information:**

The online version contains supplementary material available at 10.1186/s13068-021-02029-2.

## Background

Interest in the development of sustainable energy using eco-friendly and renewable biomass is increasing [1, 2]. Woody biomass offers economic and sustainable feedstock for bioenergy production [3–5]. While both herbaceous (e.g., grass) and woody biomass are suitable forms of plant biomass for biofuel production [6, 7], grass biomass has the advantage of high saccharification efficiency as it is composed of polysaccharides that are easily converted to bioethanol [8]. However, the use of grass biomass for bioenergy must overcome certain logistics challenges stemming from low biomass density and limited period of harvest [9]. On the other hand, woody biomass (from perennial woody plants or trees) has environmental and economic advantages compared to herbaceous biomass because it can be produced in large quantities at high density even on marginal land and can be harvested at any time during the year [6, 10]. Furthermore, as trees grow, erosion is mitigated, carbon dioxide is captured, oxygen is produced, and biodiversity is supported. In addition, trees can provide food and raw materials for human [11].

Gibberellin 20-oxidase (GA20ox) is a key enzyme involved in the biosynthesis of bioactive gibberellic acids (GAs) that influences various aspects of plant growth and development, such as stem elongation, flowering, wood formation, and bud dormancy cycle [12–16]. Overexpression of *GA20ox* has been reported to increase plant height [17–20]. However, undesirable side effects, such as poor root/leaf development and slender stems, have been reported in many plants including transgenic poplar overexpressing *GA20ox1* [21–23].

Perennial woody plants have evolved a circadian clock to synchronize their growth and development to the daily and seasonal cycles of the environment [24–27]. Bud dormancy onset relies on short-day (SD) length, low temperature, and metabolic cues in autumn [26, 28, 29]. In trees, accurate timing of growth arrest is critical for resistance to drought and/or freezing stresses in winter [30]. The onset of the growing season is marked by bud flushing in spring when days become warmer and longer. Timely completion of this important active growth-dormancy cycle is a prerequisite for survival of perennial woody plants, especially in temperate regions [13, 28]. Endogenous levels of GAs decrease during growth cessation and dormancy establishment. In poplar, GA pathways are downregulated early during growth cessation by SD photoperiod [30, 31]. Indeed, hybrid aspens overexpressing *Arabidopsis GA20ox1* were unable to arrest growth for bud set and dormancy establishment, even during the SD photoperiod, due to high level of GA [31].

To address the issue of high GA content due to 35S promoter-driven constitutive overexpression of *GA20ox1*, we utilized a developing xylem (DX) tissue-specific promoter (i.e., DX15 promoter) to express *PdGA20ox1*, a *GA20ox1* from *Pinus densiflora* [19], to avoid the undesirable phenotypes, and achieved up to threefold increased biomass production in hybrid poplars [22].

Woody biomass is primarily derived from secondary cell walls that comprise cellulose, hemicellulose, and lignin [32, 33]. Several MYB transcription factors (TF) have been identified as positive regulators of secondary wall formation, and MYB46 plays a pivotal role as a master switch for secondary wall biosynthesis in *Arabidopsis* [34, 35]. Overexpression of *MYB46* induces ectopic secondary wall biosynthesis by activating cellulose, xylan, and lignin biosynthetic genes in *Arabidopsis* [36–38]. Consistent results were reported when *PtrMYB3*, an ortholog of *MYB46* (AT5G12870) in *Populus trichocarpa*, was overexpressed [39, 40].

In an effort to improve both quantity and quality of woody biomass without unwanted growth effects, we used a bicistronic gene expression system (e.g., 2A system) to express *PdGA20ox1* and *PtrMYB221* under the DX15 promoter [41]. The 2A system allows multiple genes to be encoded in a single open reading frame with a short intervening viral 2A sequence that has self-processing properties between the coding sequences [41–43]. Because PtrMYB221 is a negative regulator of lignin biosynthesis, the resulting transgenic poplars exhibited reduced lignin content but increased biomass production [41]. We then hypothesized that the use of *PtrMYB3,* a master regulator of secondary cell wall biosynthesis, in place of the *PtrMYB221* in the previous construct design [41] might increase both overall tree growth and biomass density.

To test this hypothesis, we performed intensive analyses of the growth and biochemical characteristics of the resulting transgenic poplars grown under LMO field conditions for long periods of time to encompass different seasons. We report the findings and discuss their significance in this approach to improve woody biomass feedstock.

## Results

### Generation of transgenic *Arabidopsis* and hybrid poplars expressing both *PdGA20ox1* and *PtrMYB3*

**Fig. 1:**
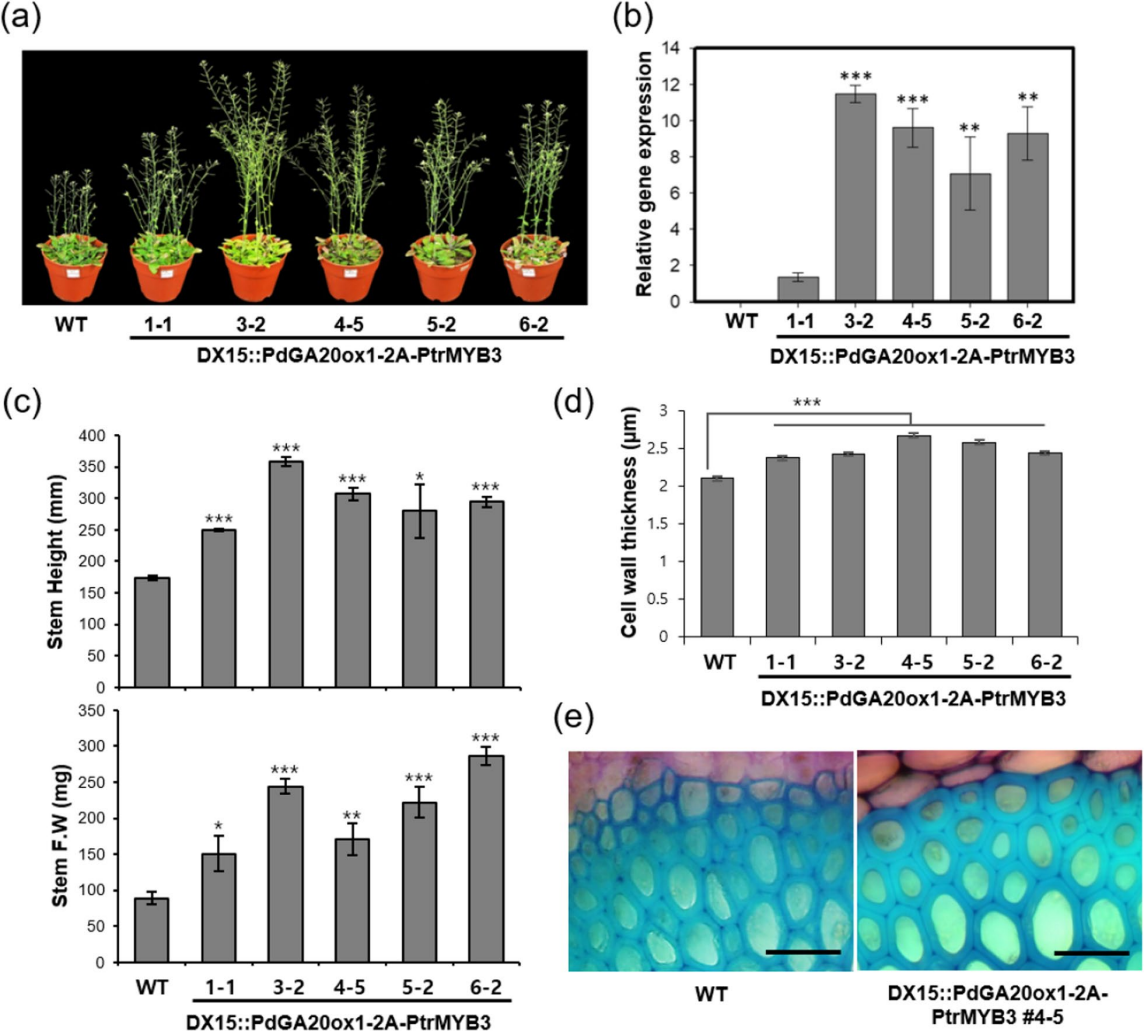
Enhanced biomass formation in transgenic *Arabidopsis* plants having DX15::PdGA20ox1-2A-PtrMYB3 construct. Growth and biomass formation were analyzed using 50-day soil-grown plants. **a** Overall growth phenotypes of transgenic *Arabidopsis* plants compared to wild-type (WT) plants. Representative photographs of five independent T3 homozygous transgenic lines (1–1, 3–2, 4–5, 5–2, and 6–2) are shown together with WT plants. **b** Expression of *PdGA20ox1-2A-PtrMYB3* transcripts in transgenic *Arabidopsis* plants. RT-qPCR was performed using cDNA templates generated from stem total RNAs. *AtActin8* was used as the internal quantitative control. **c** Increase in plant biomass in transgenic *Arabidopsis* plants compared to WT plants. Both stem length (upper) and stem fresh weight (lower) were measured. Error bars indicate S.D. (*n* = 3). **d** Quantification of cell wall thickness. Error bars indicate S.E. (*n* = 3, 150 cells per plant). **e** Histological analyses of stem tissue. Rosette level inflorescent stems from transgenic *Arabidopsis* plants (line 4–5) and WT plants were cross sectioned and stained with toluidine blue *O*. Interfascicular fiber cells were visualized. Scale bars indicate 25 µm. Unpaired Student’s *t*-test, *P*-value * (*P* < 0.05), ** (*P* < 0.01), *** (*P* < 0.001)

**Fig. 2:**
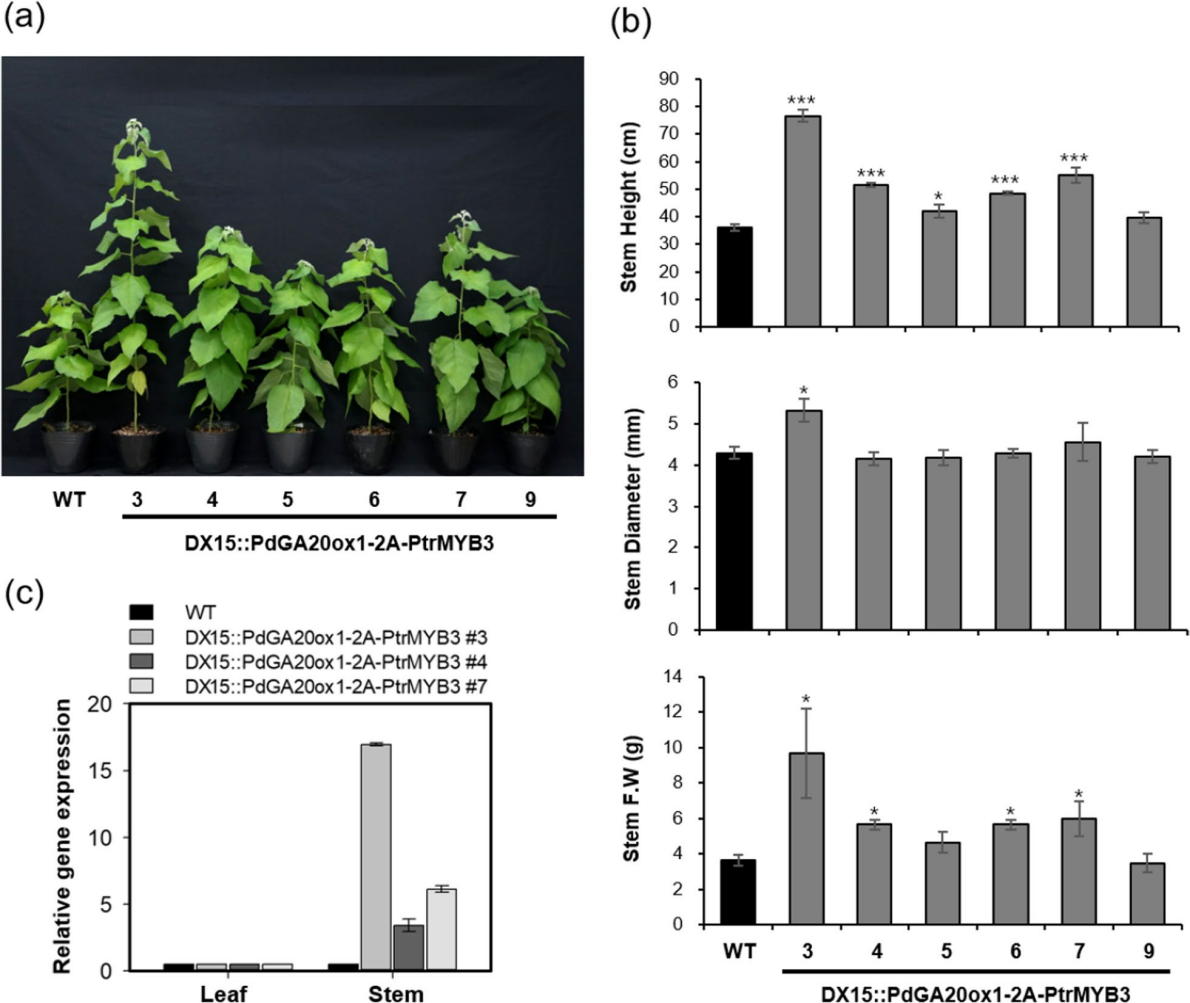
Improved growth performance of transgenic poplar plants grown in a growth room. Growth performance was analyzed using 60-day-grown poplar plants. **a** Overall growth of transgenic poplar plants (DX15::PdGA20ox1-2A-PtrMYB3) compared to WT plants. Representative photographs of six independent transgenic lines (3, 4, 5, 6, 7, 9) and WT plants are shown. **b** Improved growth of transgenic poplars. Stem height (top), diameter (middle), and fresh weight (bottom) were measured (see, methods). Error bars indicate S.D. (n = 3). **c** Expression of *PdGA20ox1-2A-PtrMYB3* transcript in transgenic poplar plants. RT-qPCR was performed using cDNA templates generated from stem total RNAs. *PtrActin2* was used as the internal quantitative control. Unpaired Student’s *t*-test, *P*-value * (*P* < 0.05), ** (*P* < 0.01), *** (*P* < 0.001)

To express both *PdGA20ox1* and *PtrMYB3* in a developing xylem (DX) tissue-specific manner, we utilized the 2A peptide sequence and DX15 promoter as reported ([41]; see methods) and produced both transgenic *Arabidopsis* and hybrid poplars (i.e., DX15::PdGA20ox1-2A-PtrMYB3 plants). For detailed phenotypic analysis, we selected five T3 homozygous transgenic *Arabidopsis* plant lines (1–1, 3–2, 4–5, 5–2, and 6–2) and six transgenic hybrid poplar lines (3, 4, 5, 6, 7, and 9) (Figs. 1 and 2). Although the DX-specific expression capacity of the DX15 promoter has been confirmed [44], we verified stem tissue-preferential expression of *PdGA20ox1* in transgenic poplar plants by both semi-quantitative RT-PCR and quantitative real-time PCR (RT-qPCR). The results showed that expression of *PdGA20ox1* was detected in the stem tissues but not in leaves (i.e., major veins removed) of the DX15::PdGA20ox1-2A-PtrMYB3 transgenic hybrid poplars (Additional file 1: Fig. S1). However, in transgenic hybrid poplars constitutively overexpressing *PdGA20ox1* (i.e., 35S::PdGA20ox1 poplar), the *PdGA20ox1* gene was strongly expressed in both stems and leaves (Additional file 1: Fig. S1).

### Enhanced biomass formation by DX-specific bicistronic expression of *PdGA20ox1* and *PtrMYB3*

Consistent with our previous findings [41], transgenic plants harboring the DX15::PdGA20ox1-2A-PtrMYB3 construct showed enhanced growth performance compared to WT plants (Figs. 1 and 2). We first confirmed an increase in transcript level of *PdGA20ox1-2A-PtrMYB3* in each T3 homozygous transgenic *Arabidopsis* line by RT-qPCR (Fig. 1b). Then we quantified biomass accumulation by measuring stem height and fresh weight of both transgenic and WT *Arabidopsis* plants (Fig. 1c). Stem height and fresh weight were dramatically increased in the transgenic *Arabidopsis* compared to the WT (up to 2- and threefold, respectively).

Consistent with the previous finding that overexpression of *Arabidopsis MYB46*, an ortholog of *PtrMYB3*, significantly increases in the thickness of the secondary walls of interfascicular fiber cells [35], our histological analysis of the stem cross sections revealed increased secondary wall thickening of interfascicular fiber cells (up to 1.27-fold) in all transgenic lines compared to WT control (Fig. 1d, e).

We measured the growth of transgenic hybrid poplars (i.e., DX15::PdGA20ox1-2A-PtrMYB3) and WT poplars grown in a growth room for 60 days (Fig. 2). As expected, stem height and fresh weight of transgenic poplars were 2- and 2.5-fold higher, respectively, than those of WT poplars (Fig. 2a, b). The amount of *PdGA20ox1-2A-PtrMYB3* transcript expressed in the stem tissue corresponded to the increase in the biomass of the transgenic hybrid poplars (Fig. 2c).

### LMO field-grown transgenic hybrid poplar trees showed increased biomass formation with minimal growth defects

**Fig. 3:**
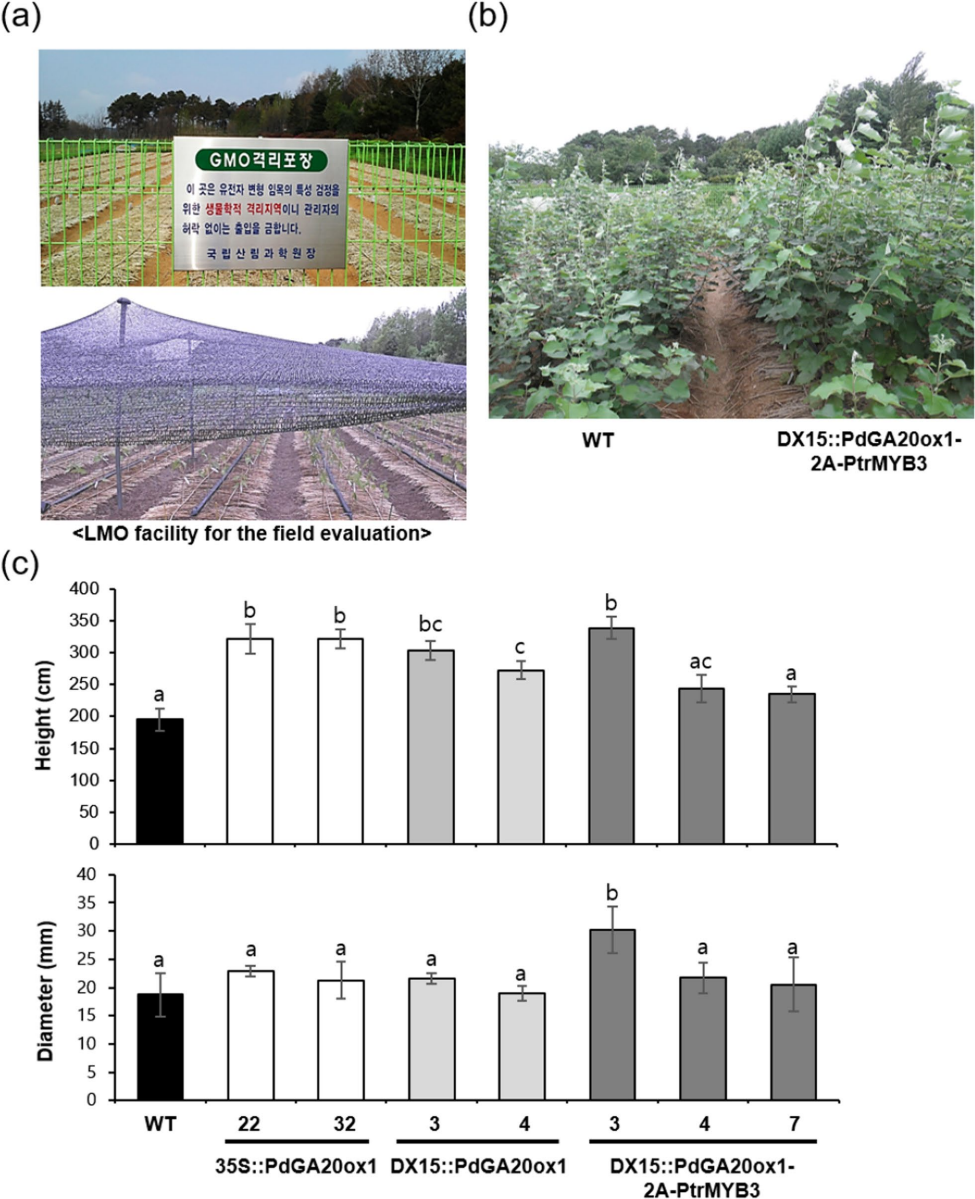
Enhanced biomass formation in transgenic poplar plants grown in an LMO field. Growth performance was analyzed using poplar plants grown in an LMO field for 3 months (from spring to summer). **a** The LMO facility used in this study (see Methods). Outside view of the facility isolated by a fence (upper panel) and shading treatment for young seedlings grown in the spring in the LMO field (lower panel). **b** A representative photograph showing the growth of WT and transgenic poplars in the LMO field in summer. **c** Measurement of stem height (upper panel) and diameter (lower panel) of transgenic hybrid poplars (i.e., 35S::PdGA20ox1, DX15::PdGA20ox1, and DX15::PdGA20ox1-2A-PtrMYB3) and comparison to these growth parameters in WT plants. Error bars indicate S.D. (*n* = 4). The same letters indicate non-significant differences among each line (one-way ANOVA with Tukey’s test, *P* < 0.05)

We evaluated the growth performance of DX15::PdGA20ox1-2A-PtrMYB3 transgenic poplars and WT poplars under LMO field conditions, along with 35S::PdGA20ox1 and DX15::PdGA20ox1 transgenic poplars, which are constitutive overexpression [19] or DX-specific expression of *PdGA20ox1* [22], respectively (Fig. 3). After 3 months of active growth in the spring to summer seasons, we measured stem height and diameter growth of the transgenic poplars to quantify biomass accumulation (Fig. 3c). The height of 35S::PdGA20ox1 and DX15::PdGA20ox1 poplars was 1.65- and 1.55-fold greater than that of WT poplars, respectively, while there was no significant difference in diameter growth. However, height and diameter growth in DX15::PdGA20ox1-2A-PtrMYB3 transgenic poplars (line 3) were increased by 1.73- and 1.62-fold, respectively (Fig. 3c). The one-way ANOVA analysis showed that the diameter growth of the DX15::PdGA20ox1-2A-PtrMYB3 transgenic poplar (line 3) is significantly increased compared to WT as well as the 35S::PdGA20ox1 and the DX15::PdGA20ox1 poplars (Fig. 3c).

**Fig. 4:**
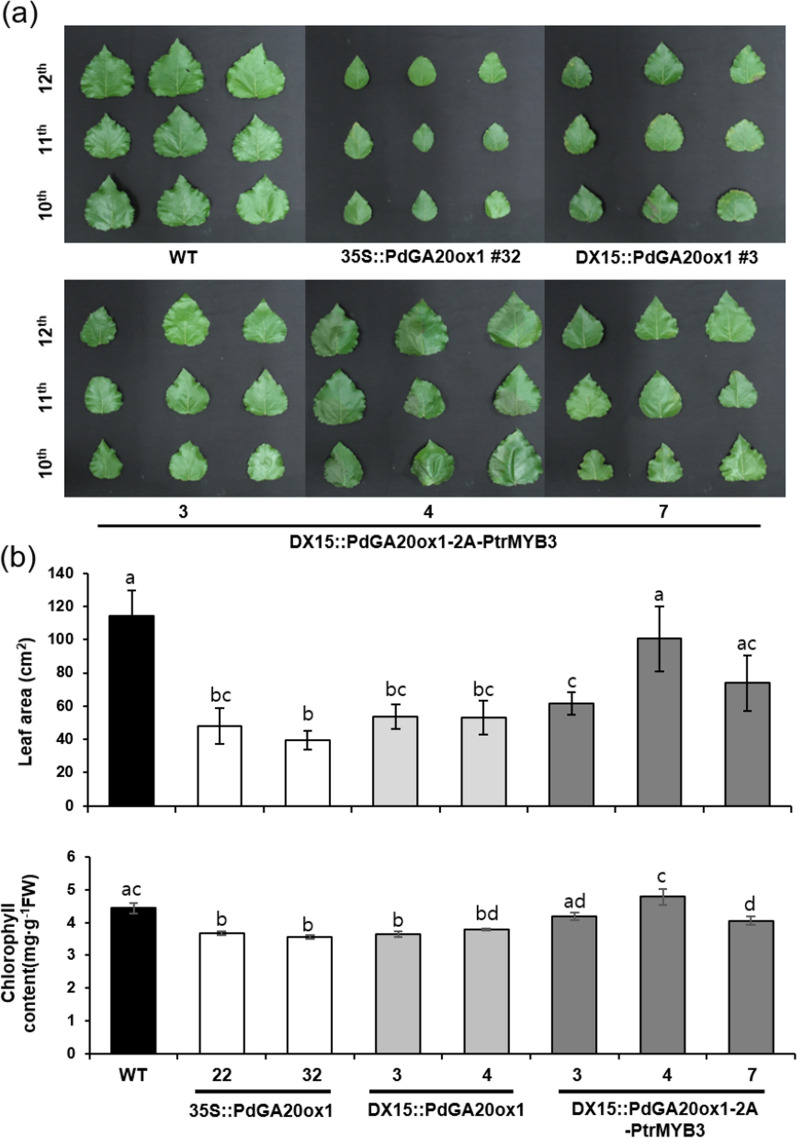
Leaf growth of transgenic poplar plants grown in an LMO field. Leaf growth and chlorophyll content were analyzed in transgenic poplar plants (35S::PdGA20ox1, DX15::PdGA20ox1 and DX15::PdGA20ox1-2A-PtrMYB3) and WT plants grown in an LMO field for 3 months (from spring to summer). **a** Representative photographs of the 10th–12th leaves from transgenic and WT poplars. **b** Measurement of leaf area (upper panel) and chlorophyll content (lower panel) in the transgenic and WT poplars shown in (**a**). Error bars indicate S.D. (*n* = 10). The same letters indicate non-significant differences among each line (one-way ANOVA with Tukey’s test, *P* < 0.05)

To assess any growth defects caused by transgenes, leaf growth was analyzed using the 10^th^–12^th^ leaves from the apex of the main stem of the transgenic poplars (Fig. 4). Both 35S::PdGA20ox1 and DX15::PdGA20ox1 transgenic poplars exhibited a 50%–65% reduction in leaf area compared to WT poplars (Fig. 4), which is consistent with a previous report [22]. However, in the case of line 4 of the DX15::PdGA20ox1-2A-PtrMYB3 transgenic poplars, leaf size and chlorophyll content were similar to those of WT poplars (Fig. 4). Taken together, these findings indicate that the DX15::PdGA20ox1-2A-PtrMYB3 transgenic poplars exhibited better growth performance under LMO field conditions than did WT, 35S::PdGA20ox1, and DX15::PdGA20ox1 transgenic poplars.

### Measurement of bud dormancy phenology and survival rates of transgenic poplars in over-winter growth analysis

**Fig. 5:**
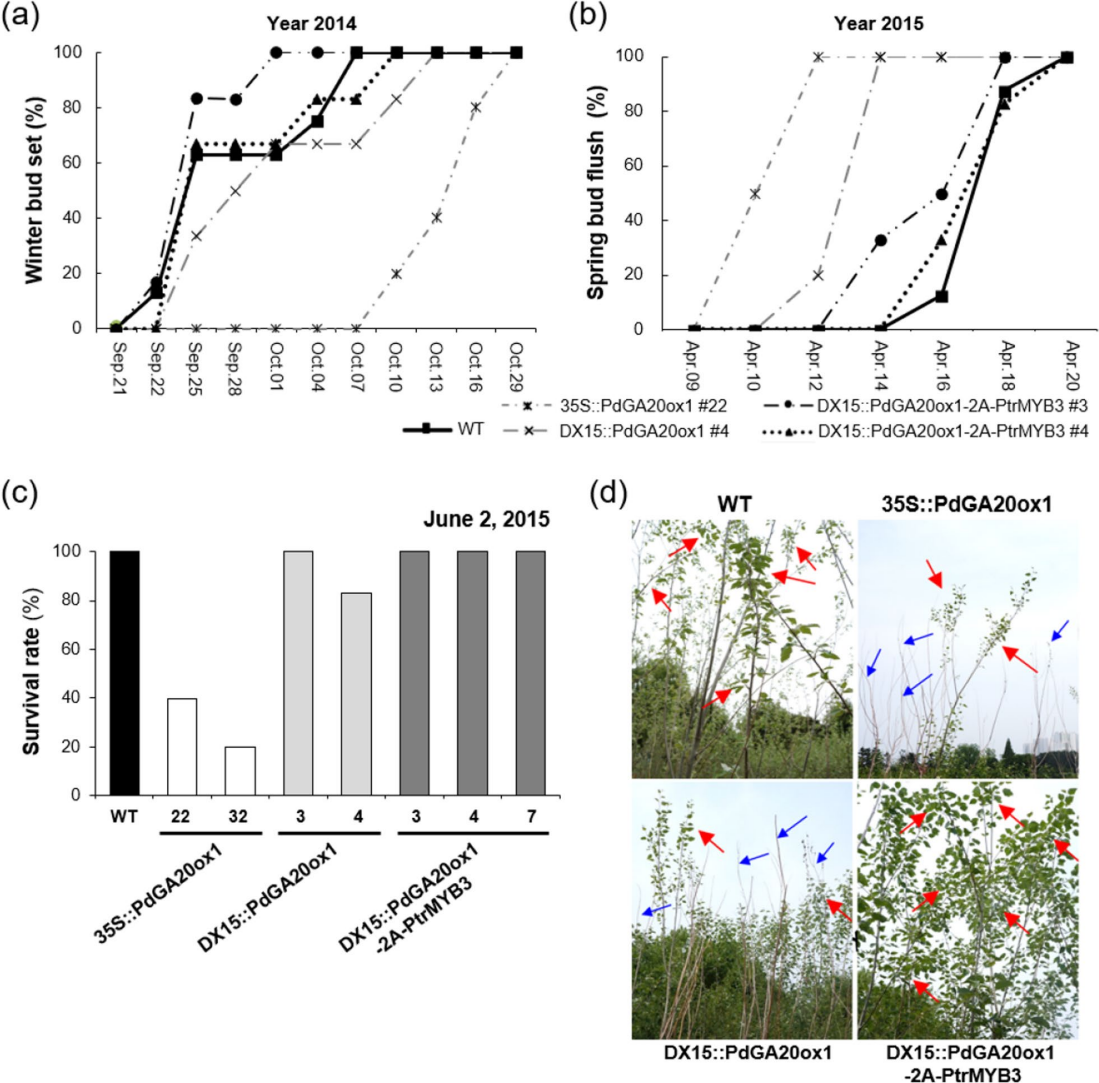
Altered bud dormancy phenology and survival rates of transgenic hybrid poplars grown in an LMO field for long periods of time encompassing changing seasons. **a** Measurement of winter bud set of transgenic hybrid poplars and WT poplars from September to October 2014. Percentage bud set was calculated from 5 to 8 trees per line. **b** Measurement of spring bud flushing of transgenic hybrid poplars and WT poplars in April of 2015. Percentage bud flush was calculated from 2 to 8 trees per line. **c** Survival rate of transgenic poplars and WT poplars after winter. Percentage survival rate was calculated from 4 to 8 trees per line in June 2015. **d** Representative photographs showing the growth of hybrid poplars in June 2015. Red arrows indicate newly emerging leaves and blue arrows show tree branches with no leaves emerging

GAs regulate the induction and release of bud dormancy in woody perennials [45–48]. Poplar species in our LMO field (see Methods) usually start winter bud set in October, and bud release occurs in April of the following year. The 35S::PdGA20ox1 and the DX15::PdGA20ox1 poplars started bud set 19 and 3 days later, respectively, than did the WT poplars. However, the bud set timing of the DX15::PdGA20ox1-2A-PtrMYB3 poplars was similar to that of WT poplars (Fig. 5a). After winter, the 35S::PdGA20ox1 and the DX15::PdGA20ox1 poplars initiated the spring bud flush 6 and 2 days earlier and completed the bud flush 9 and 6 days earlier, respectively, than did the WT poplars (Fig. 5b). However, the DX15::PdGA20ox1-2A-PtrMYB3 poplars showed a similar bud set pattern to that of WT poplars (Fig. 5b). These results suggest that the increased level of GA in the 35S::PdGA20ox1 and the DX15::PdGA20ox1 poplars altered their bud dormancy phenology. Accordingly, we observed faster shoot development from the buds of the 35S::PdGA20ox1 and the DX15::PdGA20ox1 poplars, compared to WT and DX15::PdGA20ox1-2A-PtrMYB3 poplars in the spring (Additional file 1: Fig. S2).

In over-winter growth analysis of the trees in the LMO field experiment, all of the WT and the DX15::PdGA20ox1-2A-PtrMYB3 poplars survived the winter, and leaf production by the whole plant was observed (Fig. 5c, d). By contrast, the 35S::PdGA20ox1 and the DX15::PdGA20ox1 poplars showed reduced survival rates by 20–40% and 80–100%, respectively, with significantly delayed leaf growth (Fig. 5c, d).

### Transgenic poplars produce improved quality and quantity of woody biomass

**Fig. 6:**
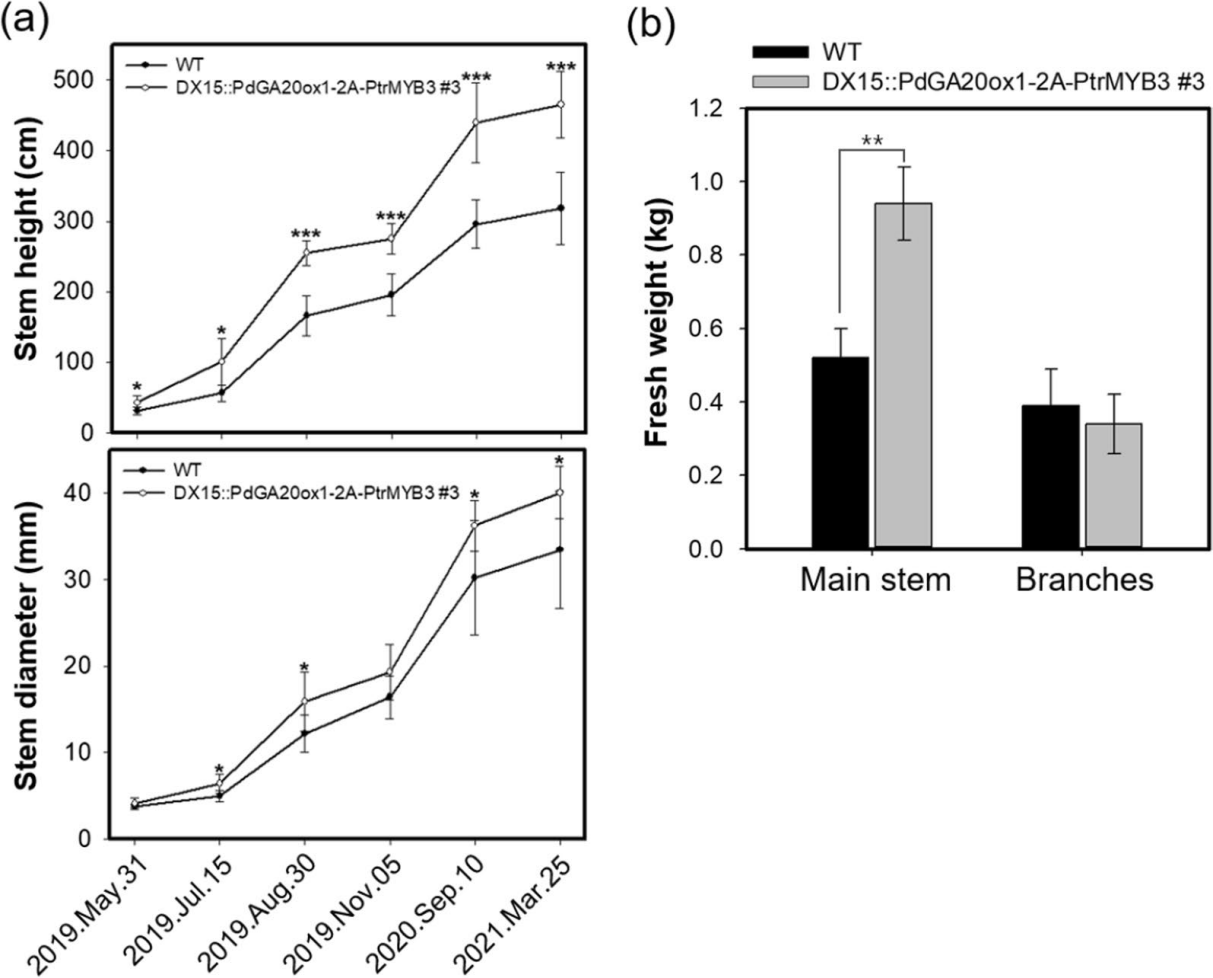
Improved woody biomass of DX15::PdGA20ox1-2A-PtrMYB3 poplars. DX15::PdGA20ox1-2A-PtrMYB3 poplars (#3) with WT hybrid poplars were grown in the LMO field for two years (May 2019 – Mar. 2021). **a** Quantification of tree growth. Stem height (upper panel) and diameter (lower panel). **b** Measurement of biomass increase. Fresh weights of main stem and branches (1^st^ and 2^nd^ branches) of each poplar were measured. Error bars indicate S.D. (*n* = 8). Unpaired Student’s *t*-test, *P*-value * (*P* < 0.05), ** (*P* < 0.01), *** (*P* < 0.001)

To validate enhanced woody biomass production in the DX15::PdGA20ox1-2A-PtrMYB3 transgenic poplars, we replanted the line 3 plants in the same LMO site in the spring and observed the plants for almost two years. As shown in the planting design (Additional file 1: Fig. S3), 26 WT and 30 DX15::PdGA20ox1-2A-PtrMYB3 poplars were planted in a total of seven beds with different combinations (Additional file 1: Fig. S3b–d). After 23 months, the DX15::PdGA20ox1-2A-PtrMYB3 transgenic poplars (line 3) had outcompeted the WT poplars (Fig. 6a, b). The stem height and diameter were 1.46- and 1.20-fold greater than those of WT plants, respectively (Fig. 6a), and fresh weight of the main stem was 1.77-fold higher (Fig. 6b). The total biomass, including the weight of the branches (first and second branches), was still significantly higher (1.41-fold) in the DX15::PdGA20ox1-2A-PtrMYB3 transgenic poplar.

**Fig. 7:**
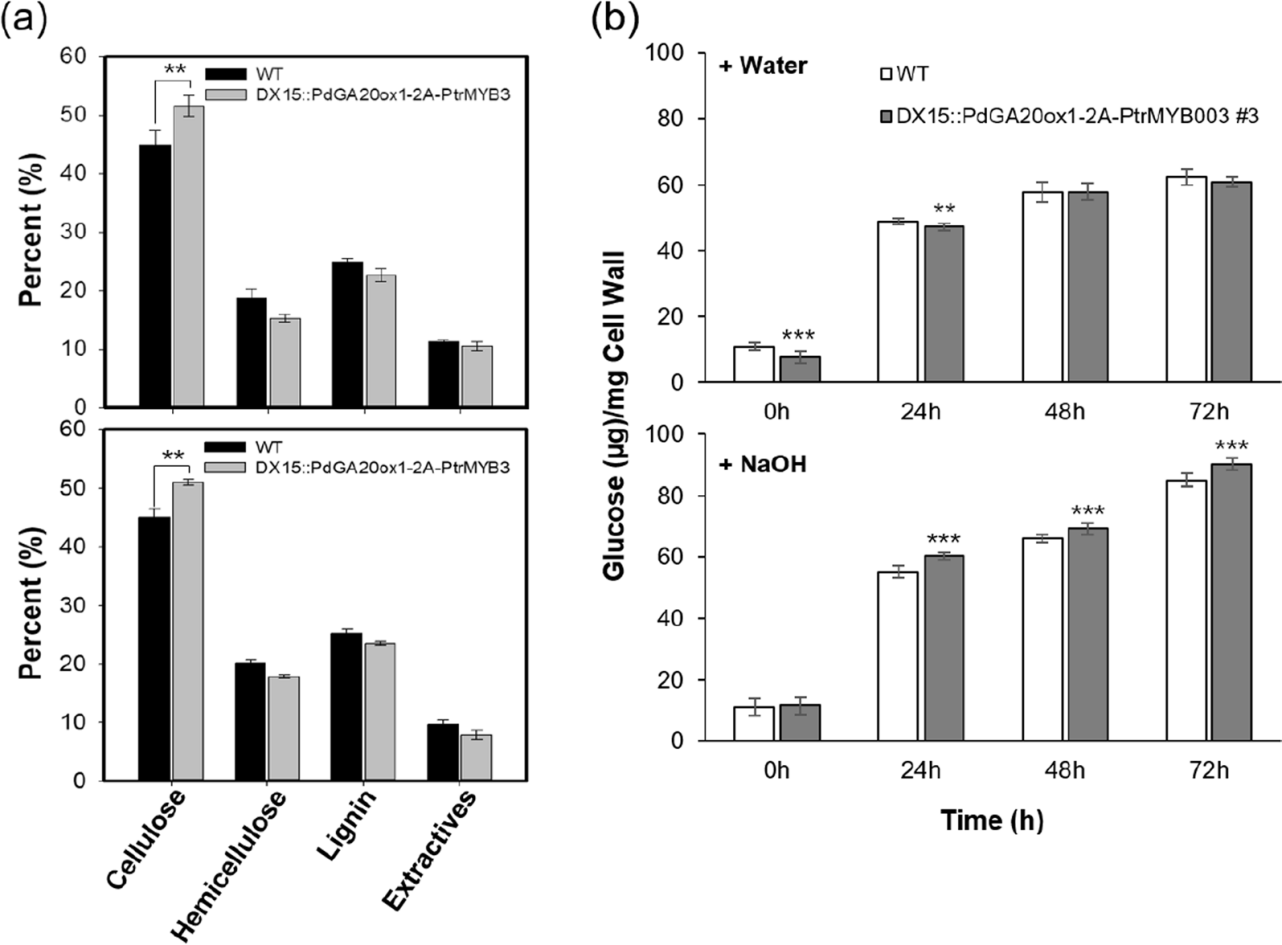
Increased saccharification efficiency of DX15::PdGA20ox1-2A-PtrMYB3 poplars. **a** Contents of cell wall components. Main stems of transgenic and WT poplars grown in LMO field for 3 months (upper panel) and 2 years (lower panel) were analyzed (see, method). Error bars indicate S.D. (*n* = 3). **b** Measurement of saccharification efficiency from WT and transgenic poplars. Saccharification efficiency was estimated by analyzing the glucose content produced by cell wall materials of woody stems of poplar plants after water (upper panel) or NaOH (lower panel) treatment for the indicated time (see Methods). Error bars indicate S.D. (*n* = 8). Unpaired Student’s *t*-test, *P*-value * (*P* < 0.05), ** (*P* < 0.01), *** (*P* < 0.001)

We examined stem tissues of the DX15::PdGA20ox-2A-PtrMYB3 poplars to examine any changes in wood formation. The stem cross section taken at the 20^th^ internode from the 60-day-old poplars showed no significant differences, compared to that from a WT poplar, including xylem cell wall thickness (Additional file 1: Fig. S4). However, the cell wall composition analysis showed a significant increase in cellulose content in transgenic poplars relative to WT poplars (up to 14.4 wt%) (Fig. 7a). Similar results were obtained from both 3-month and 2-year-old poplars grown in LMO field (Fig. 7a).

Saccharification efficiency of the wood materials from the LMO field-grown DX15::PdGA20ox-2A-PtrMYB3 poplars was estimated by quantifying the amount of glucose released at different incubation times after hot water or alkali (NaOH) pretreatment (Fig. 7b). We observed a significant increase in saccharification efficiency in the NaOH-pretreated transgenic poplars compared to WT poplars (up to 9% at 24 h), but no significant change was found after hot water treatment (Fig. 7b). These results indicate that the transgenic poplars have enhanced wood formation and improved saccharification efficiency relative to WT poplars.

## Discussion

To improve the wood and growth performance of poplars for biomass production, we expressed *PtrMYB3* in a developing xylem (DX) tissue-specific manner together with *PdGA20ox1*, bicistronically. Overexpression of *PtrMYB3* under the 35S promoter in both *Arabidopsis* and poplar results in ectopic secondary wall thickening through upregulation of the biosynthesis of cellulose, xylan, and lignin [39, 40]. As a proof-of-concept experiment, we created transgenic *Arabidopsis* plants expressing the DX15::PdGA20ox1-2A-PtrMYB3. The resulting transgenic *Arabidopsis* plants showed a significant increase in secondary wall thickening in the interfascicular fibers (up to 1.27-fold) compared to WT plants (Fig. 1d, e). In addition, expression of this construct increased the fresh weight of the stem by up to threefold (Fig. 1c). These results demonstrated the efficacy of our strategy of bicistronic gene expression of *PtrMYB3* and *PdGA20ox1*, further confirming our previous findings [41].

As shown in Fig. 2, the growth of the DX15::PdGA20ox1-2A-PtrMYB3 poplars exceeded that of WT considerably, with a 2.6-fold increase of stem fresh weight of the poplars grown in the growth room for 60 days. Next, we attempted to validate the growth room performance of the transgenic poplars under LMO field conditions after 3 months of active growth in spring and summer. The stem height and diameter of the DX15::PdGA20ox1-2A-PtrMYB3 poplars were 1.7- and 1.6-times greater, respectively, than those of WT poplars with minimal growth defects (Figs. 3 and 4). Finally, we confirmed enhanced woody biomass production by the DX15::PdGA20ox1-2A-PtrMYB3 poplars by re-planting them in the same LMO field for two years (from May 2019 to Mar. 2021) (Additional file 1: Fig. S3), resulting in 1.46- and 1.77-fold greater than those of WT plants in stem height and fresh weight, respectively (Fig. 6a, b). This is significant in that the growth room performance of the transgenic poplars was validated in actual field conditions.

It is notable that we could not find any significant histological changes in secondary wall formation in DX15::PdGA20ox1-2A-PtrMYB3 poplars, including secondary wall thickening of stem tissue (Additional file 1: Fig. S4), in light of the finding that the transgenic *Arabidopsis* plants expressing the same construct had increased secondary wall thickening of their interfascicular fiber cells (up to 1.27-fold) (Fig. 1d, e). However, in cell wall composition analysis, the cellulose content of the DX15::PdGA20ox1-2A-PtrMYB3 poplars was 14.4 wt% greater than that of WT poplars (Fig. 7a). Thus, we hypothesized that the higher content of cellulose may contribute to the increase of saccharification efficiency. However, it is not known yet why only the cellulose content was increased. Additional studies are needed to address the possibility of post-transcriptional regulation of the biosynthesis of the other cell wall components. Nonetheless, it is noteworthy that transgenic rice plants with increased cellulose content had significant increase in saccharification efficiency regardless of the changes in the other cell wall components [49].

Survival and productivity of temperate perennial woody plants depend on proper timing of dormancy onset and release, which is largely regulated by a plant hormone, GA, in many woody plants [45–48]. *GA20ox1* expression in *Populus* spp. is regulated by day length, and levels of bioactive GAs are downregulated by SD condition, by which mechanism ensures a rapid cessation of growth for bud set and dormancy establishment [11]. Indeed, hybrid aspens overexpressing *Arabidopsis GA20ox1* were unable to arrest growth even under SD conditions [31]. Previously, we reported transgenic poplars with enhanced wood formation due to constitutive or developing xylem-specific expression of the *PdGA20ox1* gene, which encodes a key enzyme involved in GA biosynthesis [19, 22, 41]. However, no intensive study has been conducted on the growth performance of them under the field condition for the entire period covering active growth-dormancy cycle.

In this study, we used three transgenic poplars, namely, 35S::PdGA20ox1, DX15::PdGA20ox1, and DX15::PdGA20ox1-2A-PtrMYB3 poplars, and their WT counterparts for evaluation of their growth performance in the LMO field condition that expands to two-growing seasons. As expected, the timing of bud dormancy onset and release (e.g., bud flush) was significantly different among these poplars (Fig. 5). The 35S::PdGA20ox1 and DX15::PdGA20ox1 poplars showed delayed bud set before winter but early bud flush the next spring compared to WT and DX15::PdGA20ox1-2A-PtrMYB3 poplars (Fig. 5). It is highly probable that the altered bud dormancy phenology was due to differences in the GA content among genotypes caused by the different *PdGA20ox1* expression. We speculate that the DX15::PdGA20ox1-2A-PtrMYB3 poplars may have adequately increased level of GA due to the significantly reduced *PdGA20ox1* transcripts in the stem tissues compared to the 35S::PdGA20ox1 poplars (Additional file 1: Fig. S1) and the DX15::PdGA20ox1 poplars. It should be noted that both the 35S::PdGA20ox1 and the DX15::PdGA20ox1 poplars were reported to have very high levels of *PdGA20ox1* expression in the stem tissues [22]. Interestingly, the significantly reduced *PdGA20ox1* expression was also found in our previous study with transgenic poplar lines expressing DX15::PdGA20ox1-2A-PtrMYB221, and the winter survival rate of this poplar was also similar to that of WT poplar [41].

For temperate perennial woody plants, timely bud set is an important protection mechanism to increase the probability of survival over winter, which is characterized by cold temperatures and abiotic stresses [13, 50]. In spring, the temperature difference between night and day is large, and the timing of bud flush has a strong influence on plant survival [51]. Our transgenic hybrid poplars, especially the 35S::PdGA20ox1 poplars, showed altered bud dormancy phenology, which might have contributed to their lower over-winter survival rate (Fig. 5d). However, the DX15::PdGA20ox1-2A-PtrMYB3 poplars showed a 100% over-winter survival rate with increased biomass up to 77% compared to WT poplars (Fig. 6), further validating our bicistronic expression strategy.

## Conclusions

The resulting DX15::PdGA20ox1-2A-PtrMYB3 poplars showed enhanced biomass production in both quantity and quality with sustained growth, which was evaluated in a field tests covering the entire active growth-dormancy cycle. Thus, our biotechnological tool can be expanded to various woody crops for production of desired multi-purpose biomass feedstock. Moreover, DX15::PdGA20ox1-2A-PtrMYB3 poplars represent a useful genetic background into which many useful traits may be stacked in order to produce a designer biomass feedstock.

## Methods

### Plant materials and growth conditions

*Arabidopsis thaliana*, ecotype Columbia (Col-0), was used in both wild-type and transgenic plant experiments. *Arabidopsis* was grown in soil in a growth room (14 h light; light intensity, 150 μmol m^−2^ sec^−1^) at 23 °C or on half-strength MS medium (Murashige and Skoog, Sigma-Aldrich) containing 2% sucrose with appropriate antibiotics for screening. Hybrid poplars (*Populus alba* × *P. glandulosa*, clone BH) were used as both WT controls and transgenic plants in this study. Plants were acclimated in soil and grown in a growth room (16 h light; light intensity, 150 µmol m^−2^ s^−1^; 24 °C) or in an LMO field at the Forest Bioresources Department of the National Institute of Forest Science, Republic of Korea (latitude 37.2 N, longitude 126.9E).

### Vector construction and plant transformation

To construct a binary vector that can drive transgene expression in a developing xylem (DX)-specific manner, we modified the pMDC32 vector [52] as follows. The 2 × 35S promoter region of the pMDC32 vector was replaced with the DX15 promoter to create the DX15-pMDC32 vector [22, 44]. Full-length cDNAs encoding *PdGA20ox1* and *PtrMYB3* (Potri.001G267300.1) were amplified by polymerase chain reaction (PCR) from cDNA of *Pinus densiflora* and *Populus trichocarpa*, respectively. A virus-derived 2A peptide sequence was used to produce a fusion construct of *PdGA20ox1* (without the stop codon) and *PtrMYB3* and inserted downstream of the DX15 promoter in the DX15-pMDC32 vector using the Gateway cloning system as described previously [41]. Vector constructs were introduced into *Agrobacterium tumefaciens* strain C58, which was used to transform *Arabidopsis* and hybrid poplar by the floral dip method [53] and leaf disk transformation-regeneration method [54, 55], respectively. All constructs used in this study were verified by DNA sequencing.

### RNA extraction and RT-PCR

Total RNAs of *Arabidopsis* were extracted using Trizol reagent (Life Technologies, Carlsbad, CA, USA) as described previously [22]. Total RNAs of poplar were extracted using the cetyltrimethylammonium bromide (CTAB) method with slight modification [56] as described [41]. In brief, fine powder from plant tissues was mixed with CTAB buffer followed by phenol:chloroform:isoamyl alcohol (25:24:1) extraction. One microgram of total RNA was reverse transcribed using Superscript III reverse transcriptase (Invitrogen) in 20 μl reaction volumes. Subsequent RT-PCR was performed with 1 μl of the reaction product as a template. Quantitative real-time PCR was performed using the CFX96TM Real-Time PCR Detection System (Bio-Rad, Hercules, CA, USA) with iQTM SYBR Supermix (Bio-Rad, Hercules, CA, USA). Poplar *Actin2* gene was used as the internal quantitative control, and relative expression was calculated by the 2^−ΔΔCt^ method [57]. Sequences are provided in Additional file 1: Table S1.

### Histology and cell wall thickness measurements

Poplar main stems (20th internode) from 60-day-old soil-grown plants or rosette level stems of *Arabidopsis* plants were used to obtain hand-cut cross sections and stained with either 0.05% toluidine blue O or 2% phloroglucinol-HCl for 1 min as described in Ref. [22]. Images of stem sections were used to measure secondary cell wall thickness of xylem vessels and fibers. Three biological replicates per line were used, and 150 cells were measured per plant.

### Growth measurements

Stem height was measured using a scale bar from the top of the plant to the soil level, and stem diameter was measured using digital calipers (Mitutoyo, Japan) at 3 cm above soil level. Both leaf area and chlorophyll content were measured in the 10–12th leaves from the top using an LI-3100 area meter (LI-COR Biosciences, Lincoln, NE, USA) and ethanol extraction method [58], respectively. Three biological replicates per line were analyzed.

### Bud dormancy phenotypic measurements

Bud dormancy phenotype at the apex of the branch was scored every day to determine the dates of bud set and bud flush from September to October and April, respectively, in all poplar plants. Bud flush was recorded when the first unfolded leaf was observed at the apex of the branch. The bud set and flush dates of each line were counted and expressed as percentages relative to those of WT poplars.

### Saccharification efficiency measurement

Saccharification efficiency of transgenic poplars grown for 3 months under LMO conditions was measured. Stem tissues were dried at 65 °C for 3 days and ground to a fine powder. Reducing sugar content was determined following the procedure described by Ref. [59] with slight modifications. Briefly, for pretreatment, ground materials (~ 2 mg) were incubated in water or NaOH (1%, w/v) at 30 °C for 30 min and then autoclaved at 120 °C for 60 min. After neutralization, 300 µl of 0.1 M sodium acetate buffer (pH 5.0) containing 40 µg of tetracycline, 10 mg cellulase, and 1 mg ß-glucosidase was added. After 24, 48, and 72 h of incubation at 37 °C with shaking (180 rpm), samples were centrifuged (13,000 rpm, 3 min), and 5 µl of the supernatant was collected for reducing sugar measurement using the DNS (3,5-dinitrosalicylate) assay [60]. Reducing sugar content was quantified by measuring the absorbance at ƛ 550 with glucose solutions as standards.

### Cell wall composition analysis

The main stems of LMO field-grown hybrid poplars (3 months and 2 years old) were used for cell wall composition analysis followed by the method described in Mottiar et al. [61]. Stem tissues were dried (65 ℃/2 weeks) and ground to a fine powder. To determine extractives amounts, 400 mg of wood power was Soxhlet-extracted using acetone for 24 h and measured the weight of extractive-free wood powder. The lignin content was quantified using the acid hydrolysis procedure for Klason lignin [62]. In brief, extractive-free wood powder (200 mg) was oven-dried at 105 ºC and weighed before treatment with 72% sulfuric acid at 20 ºC (stirring every 10 min for 2 h). Acid hydrolysis was followed in 4% sulfuric acid for 1 h in an autoclave (at 121 ºC) and the acid-insoluble lignin was determined gravimetrically of the residue remaining in medium coarseness sintered glass crucibles after filtration and oven-dried at 105ºC. For cellulose content, the standard method for alpha cellulose was used following delignification. Briefly, extractive-free wood powder (100 mg) was reacted with 20% sodium chlorite in sodium acetate buffer (60 mL/L glacial acetic acid and 1.3 g/L sodium hydroxide) at 50 ºC for 16 h and repeated for another 16 h using fresh reaction mixture. After washing with 1% acetic acid and acetone, delignified wood powder was recovered from a medium coarseness sintered glass crucible. After drying at 50 ºC, 30 mg of the recovered holocellulose was incubated with 17.5% sodium hydroxide for 30 min and another 30 min after dilution to 8.75% by adding distilled water at room temperature. After filtering and rinsing through a medium coarseness sintered glass crucible, the powder was soaked in 1 M acetic acid for 5 min and then rinsed again with distilled water. Then, the alpha cellulose content was measured gravimetrically after drying at 105 ºC.

### Statistical analysis

All experiments were performed in triplicate and repeated at least three times. The number of plants used in each experiment is indicated for each result presented. Statistical analysis was performed, and graphs were generated using SigmaPlot v12.0 (Systat Software, Inc., Chicago, IL, USA). The significance of differences between groups was calculated using Student’s *t*-test, and significance level is indicated by asterisks (*P < 0.05; **P < 0.01; and ***P < 0.001).

## Supplementary Information

**Additional file 1: Table S1.** Primers used in this study. **Figure S1.** Stem-specific expression of *PdGA20ox1* transcripts in transgenic poplar plants. Gene expression in 60-day soil-grown poplar plants was analyzed. **a** Gene expression pattern of *PdGA20ox1* by semi-quantitative RT-PCR using cDNA templates generated from either stem or leaf total RNA. **b** Quantification of *PdGA20ox1* transcripts by qRT-PCR using cDNA templates generated from stem total RNA of the indicated hybrid poplars (i.e., WT, 35S::PdGA20ox1 #22, and DX15::PdGA20ox1-2A-PtrMYB003 #3). Error bars indicate S.E. (*n* = 3). **Figure S2.** Bud flushing status of transgenic hybrid poplars and WT poplars in spring. Shoot development from winter bud was faster in 35S::PdGA20ox1 and DX15::PdGA20ox1 transgenic poplars than in WT and DX15::PdGA20ox1-2A-PtrMYB3 poplars in spring. **Figure S3.** Planting design of WT and DX15::PdGA20ox1-2A-PtrMYB3 poplars in the LMO field. **a** Satellite photograph of the LMO field at the National Institute of Forest Science, Republic of Korea (latitude 37.2 N, longitude 126.9E). Hybrid poplars were planted in the yellow box. Bed numbers are indicated right. **b** Planting design of WT (green circles) and DX15::PdGA20ox1-2A-PtrMYB3 (red circles) poplars in each bed shown in (**a**). **c** Detailed planting map of each bed shown in (**b**). **d** Photograph taken two days after planting following the design. **Figure S4.** Secondary cell wall analysis of WT and DX15::PdGA20ox1-2A-PtrMYB3 transgenic poplars. **a** Histological analyses of secondary wall formation in DX15::PdGA20ox1-2A-PtrMYB3 and WT plants. The 20^th^ internodes of stems from 60-day-old soil-grown poplar plants were used for cross-sectional analysis and stained with phloroglucinol-HCl. Scale bars indicate 25 mm. **b** Quantification of cell wall thickness. There was no difference in secondary cell wall thickness between transgenic poplar and WT plants. Error bars indicate the S.D. of the mean of three biological replicates (30 cells were measured per plant).

## Data Availability

Not applicable.
