# Supplementary material for: Field evaluation of transgenic hybrid poplars with desirable wood properties and enhanced growth for biofuel production by bicistronic expression of PdGA20ox1 and PtrMYB3 in wood-forming tissue
Source: Biotechnol Biofuels. 2021 Sep 7;14:177. doi: 10.1186/s13068-021-02029-2 (PMC8425128; doi:10.1186/s13068-021-02029-2)
Supplement: Supplementary file 1 — Additional file 1: Table S1. Primers used in this study. Figure S1. Stem-specific expression of PdGA20ox1 transcripts in transgenic poplar plants. Gene expression in 60-day soil-grown poplar plants was analyzed. a Gene expression pattern of PdGA20ox1 by semi-quantitative RT-PCR using cDNA templates generated from either stem or leaf total RNA. b Quantification of PdGA20ox1 transcripts by qRT-PCR using cDNA templates generated from stem total RNA of the indicated hybrid poplars (i.e., WT, 35S::PdGA20ox1 #22, and DX15::PdGA20ox1-2A-PtrMYB003 #3). Error bars indicate S.E. (n = 3). Figure S2. Bud flushing status of transgenic hybrid poplars and WT poplars in spring. Shoot development from winter bud was faster in 35S::PdGA20ox1 and DX15::PdGA20ox1 transgenic poplars than in WT and DX15::PdGA20ox1-2A-PtrMYB3 poplars in spring. Figure S3. Planting design of WT and DX15::PdGA20ox1-2A-PtrMYB3 poplars in the LMO field. a Satellite photograph of the LMO field at the National Institute of Forest Science, Republic of Korea (latitude 37.2 N, longitude 126.9E). Hybrid poplars were planted in the yellow box. Bed numbers are indicated right. b Planting design of WT (green circles) and DX15::PdGA20ox1-2A-PtrMYB3 (red circles) poplars in each bed shown in (a). c Detailed planting map of each bed shown in (b). d Photograph taken two days after planting following the design. Figure S4. Secondary cell wall analysis of WT and DX15::PdGA20ox1-2A-PtrMYB3 transgenic poplars. a Histological analyses of secondary wall formation in DX15::PdGA20ox1-2A-PtrMYB3 and WT plants. The 20th internodes of stems from 60-day-old soil-grown poplar plants were used for cross-sectional analysis and stained with phloroglucinol-HCl. Scale bars indicate 25 mm. b Quantification of cell wall thickness. There was no difference in secondary cell wall thickness between transgenic poplar and WT plants. Error bars indicate the S.D. of the mean of three biological replicates (30 cells were measured per plant). [file 13068_2021_2029_MOESM1_ESM.docx]

Additional file 1: **Table S1.** Primers used in this study

| No | Gene ID | Gene name | Primer | Note |
| --- | --- | --- | --- | --- |
| **For gene cloning** | | | |  |
| 1 | KC461180.1 | PdGA20ox1 | Forward_aaaaagcaggctATGGGTACTTCGACTGTGAG | attB1 partial |
|  |  |  | Reverse_gacgtcacctgcaagcttaagaaggtcgaagttaagaagctg TGGCTGGTTTCTTGAGGTGA | 2A sequence |
| 2 | Potri.001G267300.1 | PtrMYB3 | Forward_cttcttaagcttgcaggtgacgtcgagtcaaacccaggtcca ATGAGGAAGCCGGATCTAATG | 2A sequence |
|  |  |  | Reverse_agaaagctgggtAACTAAAGGTTCAAAATATT | attB2 partial |
| 3 | attB1 |  | acaagtttgtacaaaaaagcaggct |  |
| 4 | attB2 |  | accactttgtacaagaaagctgggt |  |
| **For semi-quantitative RT-PCR** | | | |  |
| 5 | KC461180.1 | PdGA20ox1 | Forward_GCAAATCACTGGCATTTTTCCTG |  |
| 6 | Potri.001G267300.1 | PtrMYB3 | Reverse_TGTGGAAGTGCTGTTGATCT |  |
| 7 | KC461180.1 | PdGA20ox1 | Forward_GTGAACAAGACAACACCTCG |  |
|  |  |  | Reverse_GCTGGTTTCTTGAGGTGAAC |  |
| 8 | AT1G49240 | AtActin8 | Forward_ATGAAGATTAAGGTCGTGGCA |  |
|  |  |  | Reverse_TCCGAGTTTGAAGAGGCTAC |  |
| 9 | Potri.019G010400.1 | PtrActin2 | Forward_GCCATCTCTCATCGGAATGGAA |  |
|  |  |  | Reverse_ AGGGCAGTGATTTCCTTGCTCA |  |

**
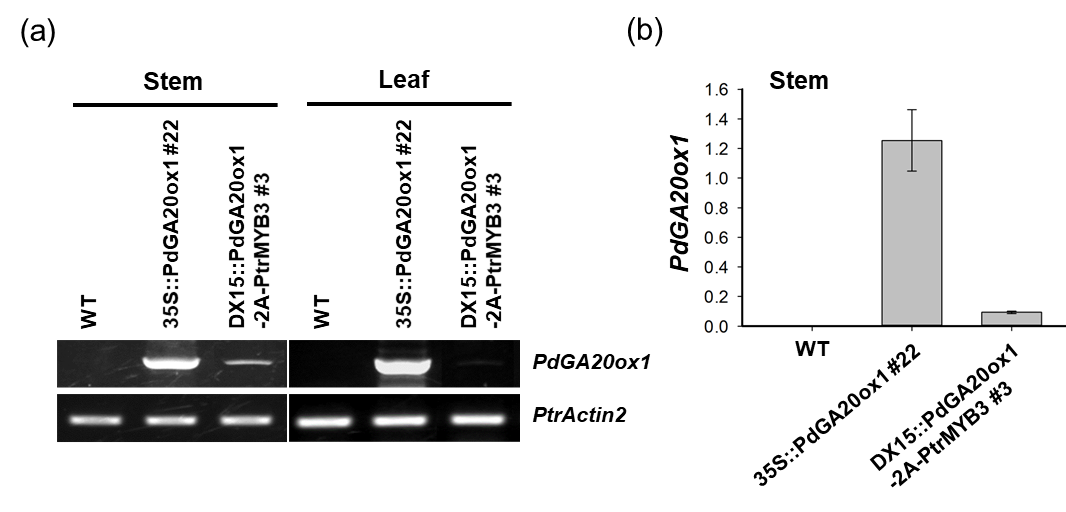
**

Additional file 1: **Figure S1. Stem-specific expression of *PdGA20ox1* transcripts in transgenic poplar plants.**

Gene expression in 60-day soil-grown poplar plants was analyzed. (a) Gene expression pattern of *PdGA20ox1* by semi-quantitative RT-PCR using cDNA templates generated from either stem or leaf total RNA. (b) Quantification of *PdGA20ox1* transcripts by qRT-PCR using cDNA templates generated from stem total RNA of the indicated hybrid poplars (i.e., WT, 35S::PdGA20ox1 #22, and DX15::PdGA20ox1-2A-PtrMYB003 #3). Error bars indicate S.E. (n = 3).


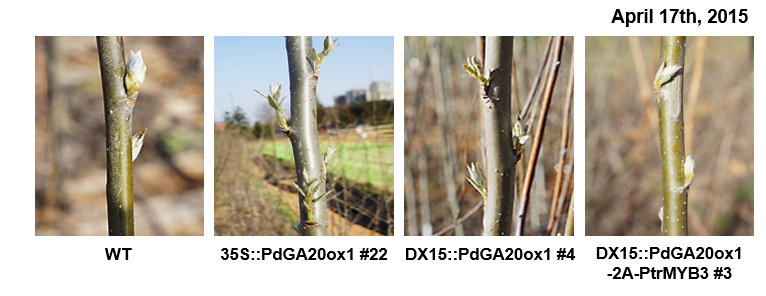


Additional file 1: **Figure S2. Bud flushing status of transgenic hybrid poplars and WT poplars in spring.**

Shoot development from winter bud was faster in 35S::PdGA20ox1 and DX15::PdGA20ox1 transgenic poplars than in WT and DX15::PdGA20ox1-2A-PtrMYB3 poplars in spring.


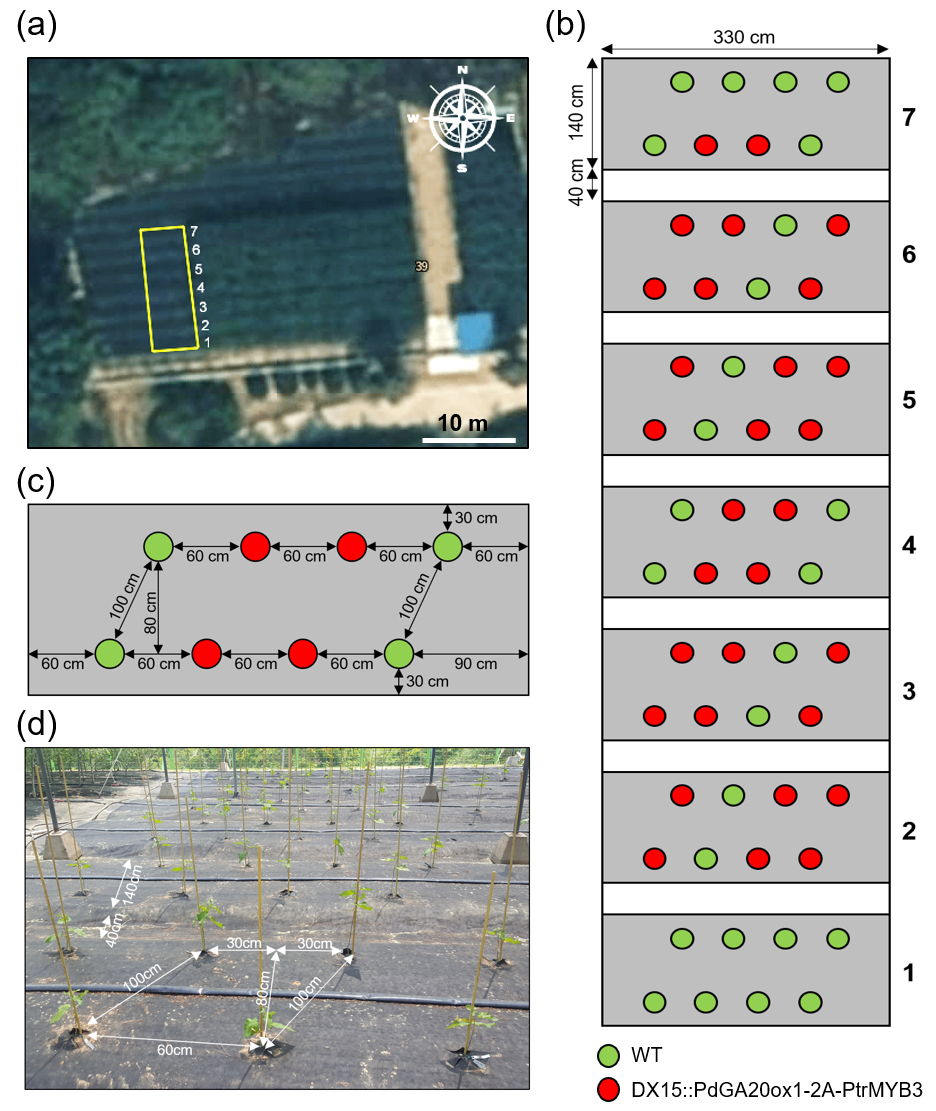


Additional file 1:  **Figure S3. Planting design of WT and DX15::PdGA20ox1-2A-PtrMYB3 poplars in the LMO field.**

(a) Satellite photograph of the LMO field at the National Institute of Forest Science, Republic of Korea (latitude 37.2N, longitude 126.9E). Hybrid poplars were planted in the yellow box. Bed numbers are indicated right. (b) Planting design of WT (green circles) and DX15::PdGA20ox1-2A-PtrMYB3 (red circles) poplars in each bed shown in (a). (c) Detailed planting map of each bed shown in (b). (d) Photograph taken two days after planting following the design.


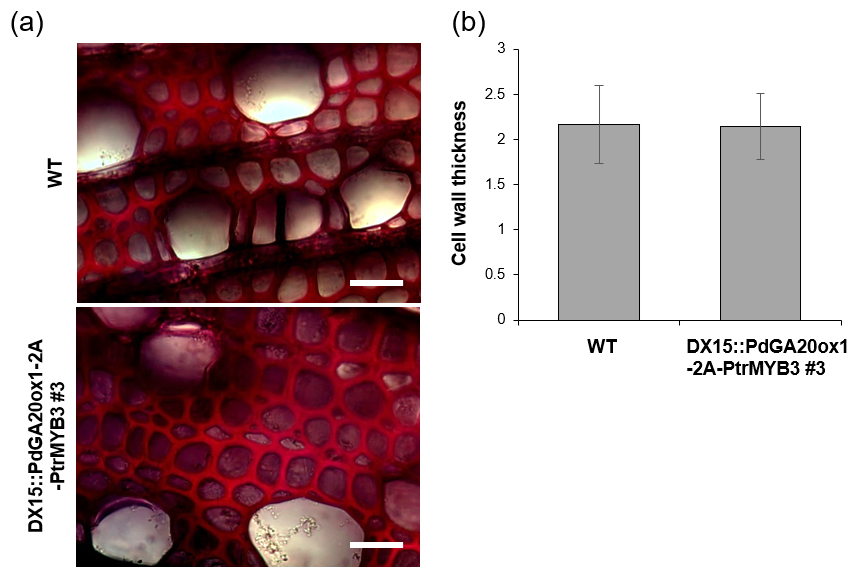


Additional file 1: **Figure S4. Secondary cell wall analysis of WT and DX15::PdGA20ox1-2A-PtrMYB3 transgenic poplars.**

(a) Histological analyses of secondary wall formation in DX15::PdGA20ox1-2A-PtrMYB3 and WT plants. The 20^th^ internodes of stems from 60-day-old soil-grown poplar plants were used for cross-sectional analysis and stained with phloroglucinol-HCl. Scale bars indicate 25 mm. (b) Quantification of cell wall thickness. There was no difference in secondary cell wall thickness between transgenic poplar and WT plants. Error bars indicate the S.D. of the mean of three biological replicates (30 cells were measured per plant).
